# Clinical benefit of range uncertainty reduction in proton treatment planning based on dual-energy CT for neuro-oncological patients

**DOI:** 10.1259/bjr.20230110

**Published:** 2023-07-26

**Authors:** Vicki Trier Taasti, Esther Decabooter, Daniëlle Eekers, Inge Compter, Ilaria Rinaldi, Marta Bogowicz, Tim van der Maas, Esther Kneepkens, Jacqueline Schiffelers, Cissy Stultiens, Nicole Hendrix, Mirthe Pijls, Rik Emmah, Gabriel Paiva Fonseca, Mirko Unipan, Wouter van Elmpt

**Affiliations:** 1 Department of Radiation Oncology (MAASTRO), GROW – School for Oncology and Reproduction, Maastricht University Medical Centre+, Maastricht, The Netherlands

## Abstract

**Objective:**

Several studies have shown that dual-energy CT (DECT) can lead to improved accuracy for proton range estimation. This study investigated the clinical benefit of reduced range uncertainty, enabled by DECT, in robust optimisation for neuro-oncological patients.

**Methods:**

DECT scans for 27 neuro-oncological patients were included. Commercial software was applied to create stopping-power ratio (SPR) maps based on the DECT scan. Two plans were robustly optimised on the SPR map, keeping the beam and plan settings identical to the clinical plan. One plan was robustly optimised and evaluated with a range uncertainty of 3% (as used clinically; denoted 3%-plan); the second plan applied a range uncertainty of 2% (2%-plan). Both plans were clinical acceptable and optimal. The dose–volume histogram parameters were compared between the two plans. Two experienced neuro-radiation oncologists determined the relevant dose difference for each organ-at-risk (OAR). Moreover, the OAR toxicity levels were assessed.

**Results:**

For 24 patients, a dose reduction >0.5/1 Gy (relevant dose difference depending on the OAR) was seen in one or more OARs for the 2%-plan; *e.g*. for brainstem D_0.03cc_ in 10 patients, and hippocampus D_40%_ in 6 patients. Furthermore, 12 patients had a reduction in toxicity level for one or two OARs, showing a clear benefit for the patient.

**Conclusion:**

Robust optimisation with reduced range uncertainty allows for reduction of OAR toxicity, providing a rationale for clinical implementation. Based on these results, we have clinically introduced DECT-based proton treatment planning for neuro-oncological patients, accompanied with a reduced range uncertainty of 2%.

**Advances in knowledge:**

This study shows the clinical benefit of range uncertainty reduction from 3% to 2% in robustly optimised proton plans. A dose reduction to one or more OARs was seen for 89% of the patients, and 44% of the patients had an expected toxicity level decrease.

## Introduction

One of the main advantages of proton therapy is the finite range which ensures healthy tissue sparing distally to the tumour.^1^ However, estimating the proton range based on conventional X-ray CT images, so-called single-energy CT (SECT), by the use of an empirical piecewise linear conversion curve^2,3^ leads to an uncertainty in the proton range estimation.^4,5^ This range uncertainty is typically accounted for by adding an extra margin or by applying robust optimisation,^6,7^ with a range uncertainty of 3–3.5% of the proton range.^8,9^ Several factors contribute to the range uncertainty,^4,8^ amongst others CT imaging artefacts,^10^ CT-based stopping-power ratio (SPR) estimation,^4^ and mean excitation energy.^11,12^

Dual-energy CT (DECT) has been shown to provide more accurate proton SPR estimation.^10,13–15^ A thorough investigation of the different contributions to DECT-based range uncertainties, including, *e.g.* CT scanner calibration and *I*-value uncertainty, was performed by Peters et al, and they found that the range uncertainty was 1.7% for neuro-oncological and 2% for pelvic patients.^16^ They implemented DECT-based SPR estimation in clinical routine and showed the benefit of this range reduction on a single patient. Li et al also looked into the size of the range uncertainty when applying DECT, and they as well found the range uncertainty to be around 2%.^17^ Furthermore, Kassaee et al have also clinically introduced DECT imaging for neuro-oncological and head-and-neck cancer patients along with a range uncertainty of 2% in their robust optimisation.^18^ The clinical benefit of range uncertainty reduction in the robust optimisation has also been investigated by re-optimising proton plans with different levels of range uncertainty for neuro-oncological patients^19^ and for head-and-neck cancer patients.^20,21^ It was further shown that the reduced range uncertainty may allow for the use of beam directions which would typically be avoided not to risk overdosage and the placement of a high linear energy transfer (LET) in organs-at-risk (OARs) located distally to the target.^22^ However, a comprehensive analysis of the clinical impact on a large cohort of patients is still missing. Furthermore, the effect of reduced range uncertainty needs to be put in a clinical context by estimating the reduction in expected treatment-related toxicity.

In this study, we investigated the clinical relevance of reducing the range uncertainty from 3% (used in our current clinical practice) to 2% (as reported in the literature to be meaningful for DECT-based SPR estimation), based on a sizable cohort of neuro-oncological patients clinically treated with proton therapy. To assess the clinical relevance, we evaluated if the potential dose reduction to the OARs led to a reduction in the expected toxicity level, based on the recently published Radiation Oncology Collaborative Comparison Group (ROCOCO) Performance Scoring System.^23^ The aim of this study was therefore to evaluate if the effort of changing clinical procedures to start using DECT for treatment planning would be justified by a clinical benefit to the patients achieved by the reduced range uncertainty.

## Methods and materials

### Patient cohort and scanning parameters

In this study, 27 neuro-oncological patients were included. The patient characteristics are listed in Table 1. The patients were all treated with proton therapy in a clinical routine workflow, with a treatment plan created on a planning SECT scan (pSECT). During their treatment, they received weekly repeat-CTs, which were acquired in both SECT (reSECT) and DECT mode. For this study, the DECT scan from the first repeat-CT was used, typically acquired after 1 week of treatment. Institutional Review Board approval was granted (W 21 03 00100).

**Table 1.** Patient characteristics

Characteristics:	
Male/Female	12/15
Age: mean (range)	50 years (28–72 years)
*Diagnosis:*	
Astrocytoma G2	7
Craniopharyngioma	1
Pituitary adenoma	2
Meningioma G1/G2	3/2
Oligodendroglioma G2/G3	6/5
Optic nerve sheath meningioma	1
*Location:*	
Frontal	11
Temporal	4
Base of skull	6
Parietal	2
Frontotemporal	2
Other (optic nerve, parieto-occipital, frontoparietal, temporoparietal)	4
CTV volume (cc): mean (range)	142.8 cc (0.6–301.3 cc)
*Prescription dose/Number of fractions:*	
50.4 Gy / 28	17
52.2 Gy / 29	1
54.0 Gy / 30	2
59.4 Gy / 33	7

*Abbreviations*: CTV – clinical target volume; G1, G2, G3 – WHO Grade 1, 2, 3.

The DECT scans were acquired in either Dual Source mode with a Siemens SOMATOM Drive scanner (Siemens Healthineers, Forchheim, Germany; 80/Sn140 kVp – Sn: 0.4 mm tin filter; simultaneous acquisition; field-of-view of 330 mm) for 15 patients, or in Dual Spiral mode on a Siemens SOMATOM Confidence scanner (80/140 kVp; sequential acquisition; field-of-view of 350 mm) for 12 patients. The DECT images were reconstructed applying a Qr40 kernel with beam hardening correction for bone and iterative reconstruction—ADMIRE (Drive) or SAFIRE (Confidence)—at strength level 3. The SECT scans were acquired at 120 kVp and were reconstructed with same reconstruction algorithm, in general the settings for the DECT scans were based on the clinical settings for the SECT scans.

### DECT post-processing

The DECT scans were converted to SPR maps using commercially available software (Siemens DirectSPR within *syngo*.via). The DirectSPR images were stored in DICOM format and had a header information similar to that of CT images, whereby the treatment planning system (TPS) accepted them as CT images. These SPR maps were imported into the TPS RayStation 11A (RaySearch Laboratories, Stockholm, Sweden). The voxel-values in the DirectSPR were scaled SPR values, similar to how CT numbers in the Hounsfield unit scale represent scaled X-ray attenuation coefficients, *i.e*. SPR_scaled_ = (SPR-1)/1000. A regular CT-to-SPR conversion curve could therefore be specified in RayStation, where the scaled SPR values were seen as CT numbers. This conversion curve was linear over the full range, and simply used to rescale the SPR values and since a conversion curve needs to be connected to a CT image to allow for dose computation in RayStation. The SPR values in the DirectSPR images are given at an initial proton energy of 100 MeV, the conversion curve in RayStation was therefore specified at this energy. The SPR estimation in the DirectSPR software is based on the method proposed by Hünemohr et al,^24^ and the mean excitation energies used in this software is calculated based on the Bragg additivity rule for both tissues and water.

### Treatment planning

Each patient had a clinical proton treatment plan created for our Mevion S250i Hyperscan system (Mevion Medical Systems, Littleton, MA, USA).^25^ The Mevion system has a dynamic field collimation, and to correctly account for the multileaf collimator Monte Carlo dose computation was used both for optimisation and final dose calculation, as well as for all dose evaluations. The Monte Carlo uncertainty for the dose computations was 1%, and the dose grid was either 0.1 × 0.1 × 0.1 cm^3^, 0.15 × 0.15 × 0.15 cm^3^, or 0.2 × 0.2 × 0.2 cm^3^ depending on the proximity of the target to small OARs. All patients were planned with full intensity modulated proton therapy (IMPT), with either three beams (*N* = 17) or four beams (*N* = 10). The number of beams and the beam angles were individually chosen for each patient based on the location and the size of the tumour. For the plans made in this study, all these settings were kept the same as for the original clinical plan, and the settings were also not changed between the two plans created in this study.

The pSECT and the reSECT were rigidly registered as part of clinical routine workflow, and the reSECT and DECT were automatically registered as these had the same frame-of-reference. The structures were rigidly copied from the reSECT to the DECT image as patient movement between the SECT and DECT acquisition was very limited because of proper patient fixation. A python script was used to copy all the plan settings (number of beams, beam angles, spot spacing, dose grid settings, objective functions, objective weights, clinical goals, etc.) from the clinical plan created on the pSECT to create a new plan on the DECT scan.

For each patient, two plans were created on the DECT image. The planning strategy used in this study followed the clinical treatment planning procedures. The first plan denoted “3%-plan” was taken as the reference plan. This plan was robustly optimised with a setup uncertainty of 1 mm, and a range uncertainty of 3%, as also used in clinical practice.^26^ For the optimisation, the same objectives were, where possible, used as for the clinical plan (which was optimised on the pSECT), but the objective weights were allowed to be changed and the objectives could be adjusted if needed to obtain a clinical acceptable plan. The clinical acceptability was judged following the clinical procedure, which is based on robust evaluation based on voxel-wise minimum and maximum dose distribution (VWmin/VWmax).^27^ For the robust evaluation, the same setup uncertainty (1 mm) and range uncertainty (3%) was used as for the robust optimisation.

When a clinically acceptable 3%-plan was obtained, this plan was copied to create a “2%-plan”. This plan had the same beam arrangement and beam settings as the 3%-plan, but the range uncertainty for the robust optimisation and robust evaluation was reduced to 2% (the setup uncertainty was kept at 1 mm). The optimisation objectives were mainly kept the same as for the 3%-plan, but again the treatment planners were allowed to change the optimisation objectives to achieve a clinical acceptable plan, and they were also allowed to change the optimisation objective weights if it was possible to reduce the OAR doses further, in accordance with the as-low-as-reasonably-achievable (ALARA) principle. In general, they were asked to plan the patient following clinical procedures, *i.e*. to individually optimise the plan for the patient by reducing the dose to the OARs as much as possible. Therefore, no restrictions were imposed on how much the planners were allowed to change the objective functions or objective function weights between the 3%-plan and 2%-plan. For each patient, the same planner created the 3%-plan and 2%-plan.

### Evaluations

**Figure 1.**
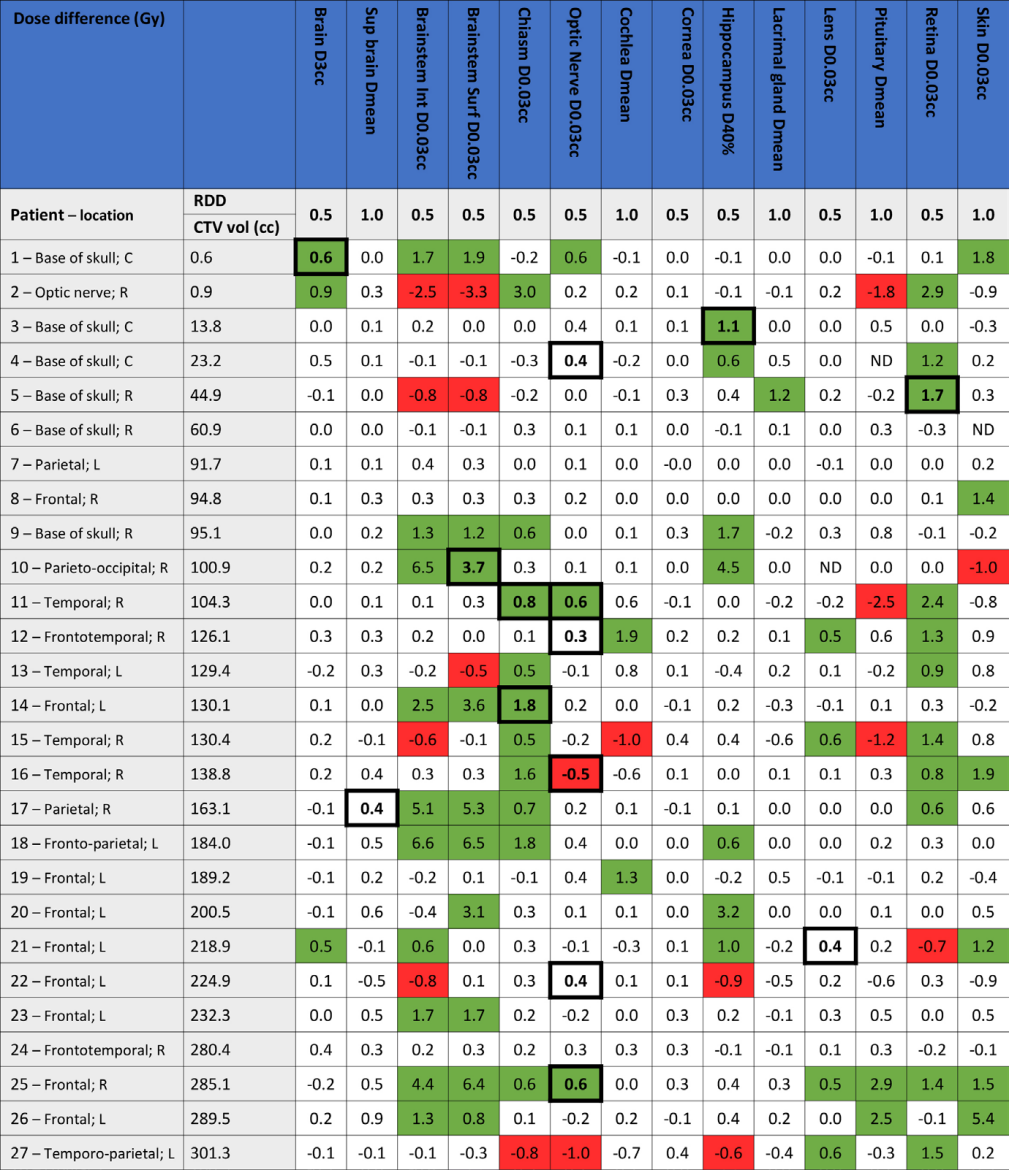
Dose differences in units of Gy, given as the dose-volume histogram (DVH) parameter for the 3%-plan minus the DVH parameter for the 2%-plan, whereby positive values indicate a benefit of the 2%-plan. The tumour location is denoted after the patient number (L: left, R: right, C: central), and the patients are sorted based on the volume of the clinical target volume (CTV). Dose differences larger than the clinically relevant dose difference (RDD; 0.5 Gy or 1.0 Gy) are marked in green (benefit of the 2%-plan) or red (disadvantage of the 2%-plan). The dose differences corresponding to a toxicity level decrease or increase are highlighted in bold and have a thick border (see details in Table 2). *Abbreviations*: Sup brain – Supratentorial brain minus CTV; Brainstem Int and Surf – Brainstem Interior and Surface (2 mm margin); ND – not delineated.

To evaluate if the 2%-plan provided clinically relevant benefits over the 3%-plan, two separate evaluations were performed. First, the dose–volume histogram (DVH) parameters for the 3%-plan and the 2%-plan were compared. Here, all DVH parameters used in clinical routine to judge if a plan is acceptable were extracted for the two plans. For each OAR DVH parameter, two experienced neuro-radiation oncologists determined which dose threshold they considered as a clinically relevant dose difference (Figure 1). The dose differences between the two plans were then assessed in relation to the dose thresholds they had specified to score potential clinical benefits of a range uncertainty reduction.

Second, to take into account that a dose difference is of less (clinical) relevance if the two doses are already far below the dose tolerance for the given OAR, the expected risk of toxicity was also evaluated by applying the ROCOCO Performance Scoring System.^23^ That is, it was assessed if a dose reduction seen for the 2%-plan would also result in a clinical benefit for the patient by leading to a reduction of the estimated risk of toxicity. This scoring system goes from 1 (lowest risk) to 5 (highest risk).

For bilateral OARs, *e.g*. the hippocampus, the DVH parameter was only reported for either the left or the right OAR, depending on which of the two got the highest dose in the 3%-plan. As done in our clinical practice, DVH parameters for mean doses (D_mean_) were extracted from the nominal dose distribution, while the maximum doses (quantified by D_0.03cc_ or D_3cc_) were extracted from the VWmax dose distribution. The target coverage was extracted from the VWmin dose distribution. All plans (both 3%-plans and 2%-plans) met the clinical acceptance criterion for the clinical target volume (CTV) V94% > 98% in the VWmin dose distribution; moreover, all OAR dose constraints were also met for all plans. The dose constraints followed the guideline published by Lambrecht et al.^28^ In addition, clinical acceptability also entails that the dose to the OARs is reduced as much as possible, in accordance with the ALARA principle, this was therefore also imposed for both the 3%- and 2%-plans.

## Results

In Figure 1, the dose differences for each patient and each OAR DVH parameter are seen. Here, dose differences larger than the clinically relevant threshold (0.5 Gy or 1 Gy, depending on the OAR) were marked. Out of the 378 dose differences (14 DVH parameters × 27 patients), 18% (*N* = 69; marked in green) were above the clinically defined threshold in favour of the 2%-plan, while only 5% (*N* = 18; marked in red) showed a clinically relevant worsening. The 18 dose differences in favour of the 3%-plan were distributed among 10 patients, but 9 of these patients had other dose differences which were in favour of the 2%-plan. For the one patient who had two DVH parameters in favour of the 3%-plan and no DVH parameters in favour of the 2%-plan (patient 22), still one dose difference in favour of the 2%-plan resulted in a toxicity level decrease, whereby the 2%-plan was preferable. 14 of the patients (52%) had one or more DVH parameters with a clinically relevant dose difference in favour of the 2%-plan, and no DVH parameters in favour of the 3%-plan, showing a clear benefit from the 2%-plan.

The data presented in Figure 1 was sorted based on the volume of CTV. However, no clear correlation between the CTV volume and the amount of positive dose differences (in favour of the 2%-plan) was seen.

**Figure 2.**
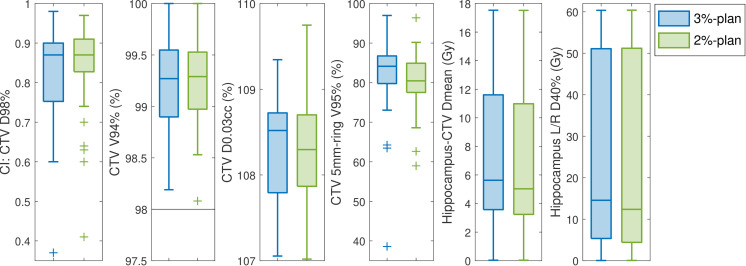
Boxplots for target conformity index (CI) and dose-volume histogram (DVH) parameters extracted from the 3%-plan (blue) and the 2%-plan (green) over all patients. The CI is calculated based on the D_98%_ for the clinical target volume (CTV) extracted from the voxel-wise minimum dose distribution (VWmin). The DVH parameters are extracted from the total dose, with prescription doses ranging from 50.4 to 59.4 Gy. The second subplot shows the target coverage, quantified by the CTV V94% extracted from VWmin; here the horizontal line at 98% denotes the minimum dose constraint. The maximum CTV dose, quantified by D_0.03cc_, is scaled to the prescription dose and extracted from VWmax. The fourth boxplot shows the V95% for a 5 mm ring structure surrounding the CTV extracted from VWmax. The fifth and sixth subplots show the mean dose to the combined hippocampus structure excluding the CTV, and the D_40%_ for the one hippocampus structure receiving the highest dose in the 3%-plan, both extracted from the nominal dose distribution. For all boxplots, the box covers the 25%- to 75%-percentile, while outliers beyond 1.5 times the interquartile range outside the box are marked with crosses.

To ensure that the OAR dose reduction was not achieved by sacrificing the target dose or the dose homogeneity to the target, we evaluated the target conformity index (CI; for CTV D_98%_), the target coverage (quantified by V94%) and the maximum dose (quantified by D_0.03cc_) for the CTV (Figure 2). It is seen that the CI is similar for the two plans (median of 0.87 for both plans). The 2%-plan also retained, or even improved the dose to the CTV, while reducing the dose outside the target. The 25%-percentile for the target coverage was slightly higher for the 2%-plan compared to the 3%-plan, while the median for the CTV maximum dose was a bit lower for the 2%-plan. The dose to the tissue right outside the CTV (5mm-ring structure) and the hippocampus dose was reduced in the 2%-plan.

12 patients (44%) had a decrease in the expected toxicity level by one level for one or two OARs, with three patients going from toxicity level 5 to 4, which was considered to be a clear clinical benefit for the patient (Table 2). Only for one patient (4%), a toxicity level increase of one level was seen for one OAR. This toxicity level increase was seen for the right optical nerve and based on a maximum dose increase of only 0.5 Gy, going from 54.4 Gy in the 3%-plan to 54.9 Gy in the 2%-plan, *i.e*. a dose increase of only 1%.

**Table 2.** Improvements and deteriorations in toxicity levels, as defined by ‘t Ven et al^23^. The third column indicates the change in the toxicity level (from the toxicity level in the 3%-plan to the toxicity level in the 2%-plan). The last column gives the dose difference (given as the dose in the 3%-plan minus the dose in the 2%-plan) corresponding to the change in the toxicity level.

Patient number	Improvements in toxicity level		Dose difference (Gy)
1	Brain (D_3cc_)	2 → 1	0.6
3	Hippocampus (D_40%_)	4 → 3	1.1
4	Optic nerve (D_0.03cc_)	4 → 3	0.4
5	Retina (D_0.03cc_)	5 → 4	1.7
10	Brainstem surface (D_0.03cc_)	3 → 2	3.7
11	Chiasm (D_0.03cc_) / Optic nerve (D_0.03cc_)	4 → 3/4 → 3	0.8/0.6
12	Optic nerve (D_0.03cc_)	4 → 3	0.3
14	Chiasm (D_0.03cc_)	3 → 2	1.8
17	Supratentorial brain minus CTV (D_mean_)	2 → 1	0.4
21	Lens (D_0.03cc_)	2 → 1	0.4
22	Optic nerve (D_0.03cc_)	5 → 4	0.4
25	Optic nerve (D_0.03cc_)	5 → 4	0.6
	**Deterioration in toxicity level**		
16	Optic nerve (D_0.03cc_)	3 → 4	−0.5

**Figure 3.**
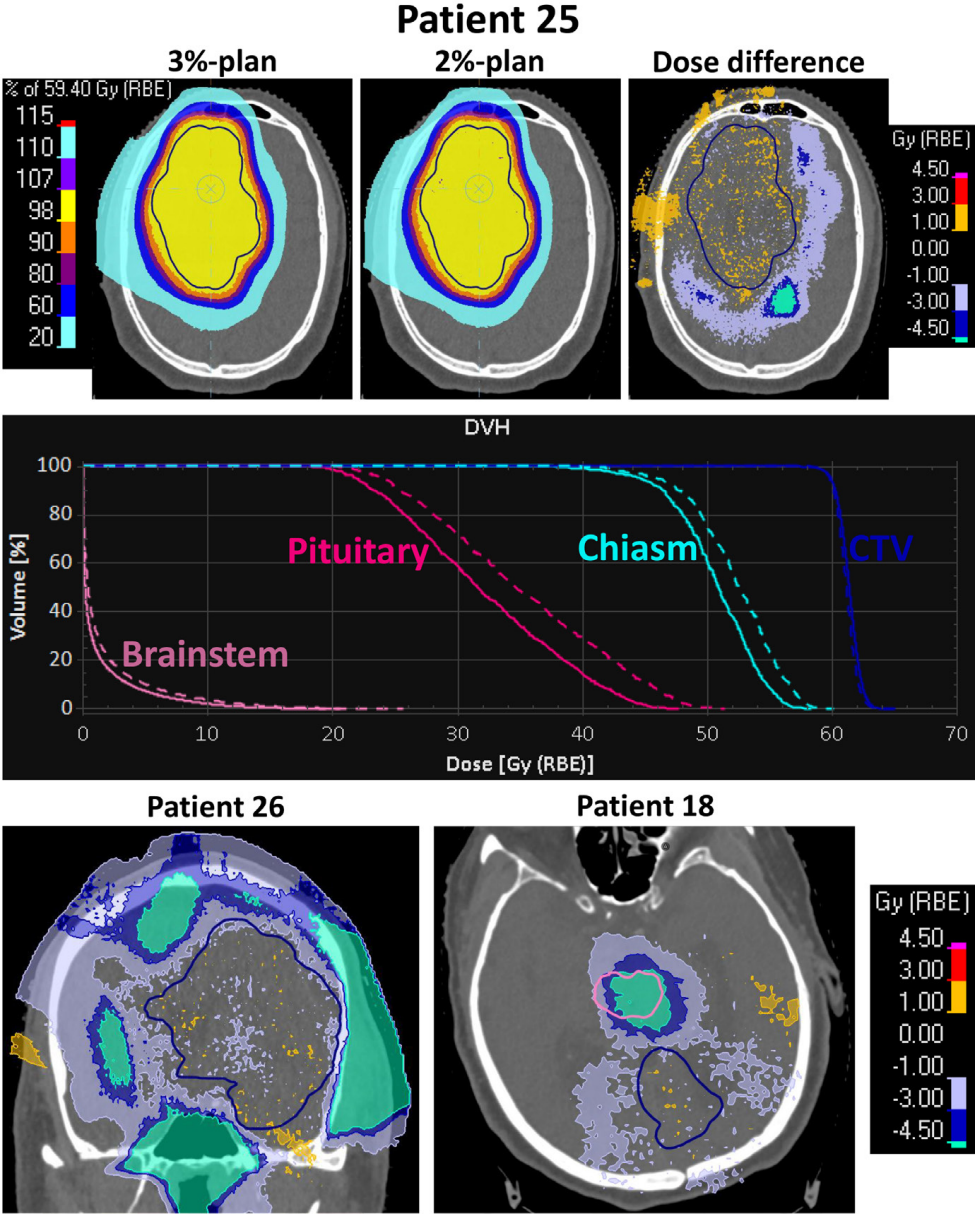
(Top) Dose distributions for patient 25 for the 3%-plan and the 2%-plan, as well as the dose difference (2%-plan minus 3% plan). The colour bar to the left of the 3%-plan is for both the 3%-plan and the 2%-plan. (Middle) Dose-volume histogram (DVH) curves for Patient 25 for the 2%-plan (full lines) and the 3%-plan (dashed lines) for organs-at-risk with large dose differences as well as for the clinical target volume (CTV). (Bottom) Dose difference for Patient 26 (coronal view) and for Patient 18. For Patient 26, large dose differences are seen all around the CTV (dark blue contour). For Patient 18, a large dose difference is seen in the brainstem (purple contour).

In Figure 3, the dose difference for three patients are seen. For the patient with eight OARs with a dose difference of a clinically relevant size (Patient 25, Figure 1), the nominal dose distribution for the 3%-plan and 2%-plan as well as the dose difference are shown for a single axial slice centrally in the CTV region. For this patient, a clear dosimetric advantage of the 2%-plan in comparison to the 3%-plan is seen at the end of the proton beam range, with dose differences above 4.5 Gy. The DVH curves are also shown for the CTV and a few OARs. The target coverage stays the same while the OAR doses decreases for the 2%-plan. Dose difference distributions are shown for two additional patients with clear gains from the 2%-plan.

## Discussion

The aim of this study was to quantify the clinical benefit proton treatment planning with a reduced range uncertainty for neuro-oncological patients. Introducing DECT in the clinical workflow allows for a reduction in range uncertainty which is not possible using SECT-based SPR estimation methods. Although, it might be expected that a range uncertainty reduction would lead to a dose reduction, it is not clear if such a dose reduction will also lead to a clinical benefit by a reduced toxicity probability. A reduction in the expected toxicity while keeping the target coverage similar is one of the main goals of treatment planning.

There are several contributing factors to the proton range uncertainty,^4,8,16^ such as CT acquisition settings and artefacts,^10,16^ mean excitation energy^11,12^ and SPR estimation.^4^ For an overview of the different contributions to the SPR uncertainty, see amongst others the following references.^4,8,16,17,29,30^ Utilising DECT to reduce the range uncertainty is one of the ways to improve the treatment planning strategy. Based on the results presented in this study, as well as the evidence in the literature, it is seen that a DECT-based treatment planning workflow is clinically feasible in clinics nowadays. Based on this, we have introduced DECT clinically for our neuro-oncological patients using the DirectSPR algorithm along with a reduced range uncertainty of 2%.

We observed a reduction in the expected toxicity level for 44% of the patients, which was deemed clinically relevant. However, since the 2%-plan was a new treatment plan optimisation compared to the 3%-plan, there might be the possibility for trade-offs between different OARs which is inherently always the case for reoptimisation, as optimisation is not a deterministic problem. This explains why for some patients, a dose increase was seen for some OARs in the 2%-plan, while a dose reduction was seen for other OARs. However, these dose differences were often small, and mainly seen for the maximum doses (D_0.03cc_) which could be caused by only a small volume having a higher dose in the 2%-plan. For example, for all three patients shown in Figure 3, the dose decrease (blue and turquoise overlay) dominated but the patients also had small areas with dose increase (orange overlay).

From Figure 1, it is seen that the DVH difference did not always correlate with a toxicity level change. For some patients, large DVH differences were seen without any toxicity level change, *e.g*. four patients had a dose reduction >5 Gy to the brainstem without a toxicity level decrease, while other patients had dose reduction of only 0.3 Gy which led to a toxicity level decrease. This could occur if the DVH parameter extracted from the two plans was close to the defined threshold for the toxicity level.^23^ For this reason, we included both a DVH-based and a toxicity level-based evaluation.

To have a fair comparison between the two plans,^31^ the optimisation objectives were as much as possible kept the same for the two plans. The optimisation objectives for the 3%-plan was based on the clinical plan, and the optimisation objectives for the 2%-plan was based on the 3%-plan. However, to ensure that both the 3%-plan and 2%-plan fulfilled the ALARA principle, the treatment planners were allowed to adjust the optimisation objectives for the 3%-plan and the 2%-plan, to have as much OAR sparing as possible in both plans. In 7/27 patients, there were no changes to the optimisation objective functions between the two plans. For seven patients, the weight of one or more of the objective functions had been changed, while for 13 patients, new objective functions had been added, removed and/or changed in the optimisation of the 2%-plan. Examples of changes to the objective functions included decreasing the maximum dose constraint for the CTV, decreasing the distance for dose fall-off objective functions, increasing/decreasing maximum dose constraints for an OAR, or increasing/decreasing the maximum equivalent uniform dose (EUD) for an OAR. New help structures and objective functions for these could also be introduced in the optimisation of the 2%-plan, while some objective functions applied for the 3%-plan could also be removed in the optimisation of the 2%-plan, especially for maximum dose help structures used for the 3%-plan. In general, the optimisation of both the 3%-plan and the 2%-plan followed the clinical trial-and-error optimisation procedure used to obtain a plan fulfilling all dose constraints, and with the best possible target coverage and lowest possible OAR dose. For some patients, the only changes were to push harder on the dose to the OARs, while for other patients even objective functions for the target had been changed in order to achieve a clinically acceptable plan for the 2%-plan. This mainly happened for challenging plans, were many OAR objective functions were included and where several optimisation help structures had to be introduced. For such plans, it would not have led to a fair comparison if the optimisation objectives had not been allowed to be changed between the two plans, since this would have led to suboptimal plan for the 2%-plan. For the simple plans, where there was room to spare the OARs additionally with the 2%-plan, this was done even though this makes the comparison less straightforward. We admit that allowing for changing the objection functions/objection function weights between the two plans, this study is not only a comparison of using a range uncertainty of 3% or 2% in the robust optimisation. But as the aim was to investigate the clinical benefits of a range uncertainty reduction, this was allowed for, since in a clinical routine the treatment planners would also always try to obtain the best possible plan even for difficult geometries.

Automated treatment planning strategies, including multi-criteria optimization, which ensures Pareto optimality can be used to ensure that treatment plans are achieved with the lowest dose to the OARs.^32–36^ However, such optimisation strategies are currently not available for proton treatment planning for the Mevion proton machine.

The patient cohort in this study was very diverse with a variety of tumour types and locations (Table 1). The benefit of a range uncertainty reduction will depend on the tumour location (beam path and nearby OARs) as the dose reduction will be seen at the end of the proton range. We therefore did not see one OAR which clearly benefitted for all patients due to the mixed cohort. However, as this patient mixture is representing the clinical reality at our proton clinic, we wanted to see the results for the general population of patients instead of only including one specific tumour type. Only one patient had a toxicity increase (Patient 16) with the 2%-plan caused by a dose difference of 0.5 Gy (1% dose increase), but this patient also had a 1.6 Gy dose decrease to the chiasm. One other patient had a clinically relevant dose increase for the maximum dose to the brainstem interior and D40% for the hippocampus (Patient 22), but at the same time this patient had a toxicity level decrease for the optic nerve. Therefore, for no patient, the 2%-plan was exclusively worse than the 3%-plan. In contrast, 44% of the patients had a toxicity level decrease, which was seen as a clear benefit of the 2%-plan. We therefore concluded that the range uncertainty reduction down to 2% was beneficial for all proton neuro-oncological patients at our clinic.

Several studies have shown the improved SPR estimation accuracy of DECT in comparison to SECT to be of an order which would justify a range uncertainty reduction from 3 to 2%.^14,37–39^ Peters et al found the range uncertainty to be 1.7–2% when using a well-calibrated DECT-based SPR estimation method.^16^ Therefore, based on the literature, the benefit of a proper validated DECT-based SPR estimation algorithm allows for a range uncertainty reduction from 3 to 2%. Tattenberg et al assessed the influence of different levels of range uncertainty (0–5%).^19,22^ We only compared two range uncertainties: the clinically used (3%) and the level suggested to be feasible when using a DECT-based treatment planning workflow (2%), as our main aim was to assess the clinical benefit of a range uncertainty of 2% (instead of 3%) made possibly by the introduction of DECT (replacing SECT).

The relative biologic effect (RBE) has often been correlated with the dose-averaged LET.^40–42^ The LET has a peak slightly beyond the Bragg peak.^43^ Reducing the dose beyond the tumour, by decreasing the range uncertainty applied in the robust optimisation, therefore could also have the opportunity to reduce the RBE to the healthy tissue beyond the tumour. However, clear relationships between RBE and LET can be hard to establish.^44,45^ Furthermore, LET calculation and reporting is not yet standardised,^46–48^ and in our clinical practice, the dose distributions are still exclusively used to judge the quality of the treatment plan. We calculated the LET distribution for a few of the patients (data not shown) but did not observe any large changes in LET distribution between the 3%-plan and 2%-plan.

Today, several different DECT scanning modes are offered on different commercial scanners, but most DECT modes have some limitations for different patient groups.^49^ However, for neuro-oncological patients, most DECT modes can be used.^37,50,51^ In this study, we used two different DECT scanners, one with sequential acquisition (and thereby, a temporal separation between the two scans) and one with simultaneous acquisition (but with a limited field-of-view of 33 cm). Both of these two limitations are of minor importance for neuro-oncological patients, which fit within the limited field-of-view and where no large movement is expected between the two scans, since the patients are fixated in a mask and no internal movement is expected in the brain.

In this study, we showed a benefit of a reduced range uncertainty resulting in a dose decrease to the OARs. This range uncertainty reduction was feasible by applying appropriate CT reconstruction settings and a well-calibrated DECT-based SPR estimation method. For proton as well as photon therapy, DECT can also be beneficial for delineation.^52,53^ By applying so-called pseudo-monoenergetic images (PMIs)^54^ at different energies, several advantages can be obtained from a single DECT acquisition, since PMIs at low energies will improve the image contrast while high energies can reduce streak and beam hardening artefacts caused by, *e.g.* metals.^10,55^

## Conclusions

We have shown that a clinical introduction of DECT-based treatment planning using a reduced range uncertainty of 2% will have a positive impact on neuro-oncological patients treated with proton therapy. In this study, 12/27 (44%) had a toxicity level decrease using the 2%-plan compared to the 3%-plan, which justified the implementation in our clinical routine. It is therefore expected that a large number of patients will benefit from the clinical implementation of DECT and such strategies need to be broadly implemented in clinical routine.
